# Anisotropic Photonics Topological Transition in Hyperbolic Metamaterials Based on Black Phosphorus

**DOI:** 10.3390/nano10091694

**Published:** 2020-08-28

**Authors:** Zengping Su, Yueke Wang

**Affiliations:** School of Science, Jiangnan University, Wuxi 214122, China; zpsu622@163.com

**Keywords:** black phosphorus, hyperbolic metamaterials, photonic topological transition, anisotropy, angular optical transparency

## Abstract

Based on in-plane anisotropy of black phosphorus (BP), anisotropic photonics topological transition (PTT) can be achieved by the proposed hyperbolic metamaterials structure, which is composed of alternating BP/SiO_2_ multilayer. Through effective medium theory and calculated iso-frequency contour, PTT can be found by carefully choosing the incident plane and other parameters. With the finite element method and transfer matrix method, a narrow angular optical transparency window with angular full width at half maximum of 1.32° exists at PTT. By changing the working wavelength, thickness of SiO_2_, or electron doping of black phosphorus, the incident plane of realizing PTT can be modulated, and anisotropic PTT is achieved.

## 1. Introduction

Metamaterials, a kind of artificial structured composites on subwavelength scales, show unprecedented electromagnetic properties which are never observed in natural materials [1]. With the development of nanofabrication techniques, the researches on metamaterials have attracted much attention, and many interesting phenomena and applications have been proposed, such as negative refraction [2,3], sub-diffraction imaging [4,5,6], metamaterials absorber [7,8,9], tunable index metamaterials [10], and biosensors [11,12]. Among the emerging varieties of metamaterials, hyperbolic metamaterials (HMMs) have rapidly drawn great attention in recent years due to their highly anisotropic features enabling hyperbolic iso-frequency dispersion and lithography-free ease of fabrication using thin film deposition methodologies [13,14]. Generally, HMMs can be constructed with metal nanowire arrays or multilayer structures of alternating metal and dielectric. In contrast to the closed iso-frequency contour (IFC) of traditional materials, the hyperbolic IFC is open and large wave-vectors can be supported in HMMs [15]. This special feature has been explored for many prospective applications in hyperlens [6], spontaneous emission enhancement [16,17], Goos–Hänchen effect [18,19], photonic topological transition [20,21,22,23,24], photonic spin hall effect [25,26], and Casimir force [27,28].

Recently, atomically thin two-dimensional (2D) materials, such as graphene, black phosphorus (BP), and hexagonal boron nitride (BN), have shown many extraordinary electronic and photonic properties [29]. As 2D materials have developed into a research hotspot, HMMs based on 2D materials has gradually attracted wide attention. As one of the most popular 2D materials, graphene can replace the role of metal in HMMs of the multilayer structure due to negative permittivity in infrared region [30]. Graphene-based HMMs have been extensively investigated in radiative heat transfer [31], negative refraction [32], slow light effect [33], and perfect absorber [34]. Compared with graphene, BP also presents metallic behavior with negative permittivity in mid-infrared region. Differently, BP have strongly inherent in-plane anisotropy, and many interesting applications have been widely explored based on this property, including anisotropic acoustic plasmons [35], polarization sensitive resonators [36], photonic spin hall effect [37], and chirality [38]. In addition, researches on BP-based HMMs has also been reported in anisotropic absorber and biaxial HMMs [39,40], but this has yet to be fully investigated. Particularly, BP-based HMMs have not been used to realize anisotropic photonic topological transition (PTT).

In this work, we theoretically propose to construct a BP-based HMMs structure consisting of alternating BP/SiO_2_ multilayer, which can realize anisotropic PTT thanks to inherent in-plane anisotropy of BP. The results obtained by finite element method (FEM) simulations and transfer matrix method (TMM) both demonstrate that a narrow angular optical transparency window can be achieved at PTT. And the structure’s IFC can transit from open hyperboloid to closed ellipsoid by changing the angle *φ* of the incidence plane. When the working wavelength *λ* = 5 μm, the *φ* of incident plane where PTT appears can be modulated from 0° to 61° by changing electron doping of BP and can be modulated from 79° to 0° by changing the thickness of SiO_2_. In addition, the angle *φ* for PTT can be modulated from 30° to 56° by changing the working wavelength. Our findings pave a new way in anisotropic angle-dependent optical applications.

## 2. Design and Theories 

**Figure 1:**
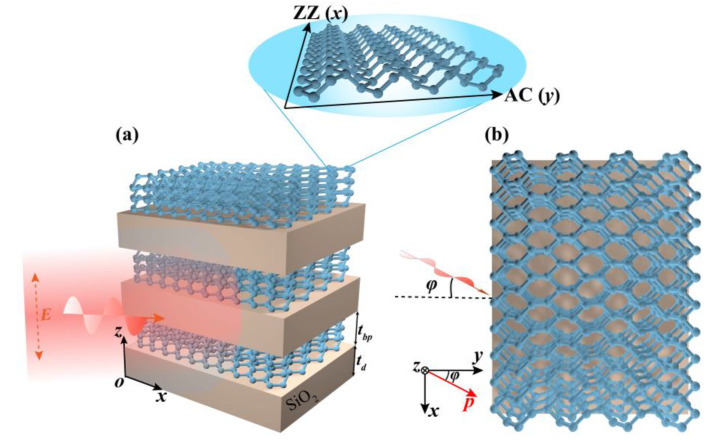
(**a**) Schematic of the proposed BP-based HMMs structure consisting of alternating BP/SiO_2_ multilayer. The thickness of the SiO_2_ and BP layer are *t_d_* and *t_bp_*, respectively. The thickness of periodic unit of the BP-based HMMs structure is *d* (= *t_d_* + *t_bp_*). (**b**) The top view of (a). A *p*-polarized light in *p*-*z* plane is incident on the side of the proposed BP-based HMMs structure from air. *φ* is the angle between the incident *p*-*z* plane and *y*-*z* plane.

As shown in Figure 1a, the proposed BP-based HMMs structure consists of periodic multilayer of BP and SiO_2_ layers, which is surrounded by air. The top view of Figure 1a is shown in Figure 1b. As depicted in Figure 1, a *p*-polarized light at wavelength *λ* is incident on the side of the proposed BP-based HMMs structure. Here, incident plane is the *p*-*z* plane, and the angle between the *p*-*z* and *y*-*z* plane is *φ*. The thickness of SiO_2_ is *t_d_* = 300 nm. The thickness of BP is given by *t_bp_* = *n* × *a_z_*/2, where *n* (= 3) is the number of layers of BP and *a_z_* (= 10.7 Å) is lattice constant in the out-of-plane direction [41]. The permittivity of SiO_2_ is 1.82. *d* (= *t_d_* + *t_bp_*) is the thickness of periodic unit of the BP-based HMMs structure. As shown in the inset of Figure 1a, the atoms in monolayer BP are covalently bonded to form a puckered honeycomb structure which leads to unique in-plane anisotropic optical property. In our proposed BP-based HMMs structure, the *x* and *y* directions correspond to the zigzag (ZZ) and armchair (AC) directions of BP, respectively. For BP, translational symmetry is broken in the *z* direction, and it has a direct energy gap at the *Г* point [41]. Thus, a low-energy in-plane Hamiltonian is used to describe the systematic behavior around the *Г* point based

(1) $$\hat{H}=\left(\begin{array}{cc} E_{c}+\eta_{c}k_{x}^{2}+\upsilon_{c}k_{y}^{2} & \gamma k_{x}+\beta k_{y}^{2}\\ \gamma k_{x}+\beta k_{y}^{2} & E_{\upsilon }-\eta_{\upsilon }k_{x}^{2}-\upsilon_{\upsilon }k_{y}^{2} \end{array}\right)$$

where *γ* and *β* describe the effective couplings between the conduction and valence bands. *E_c_* and *E_υ_* are the first conduction and valence band edge energies in BP. Near the *Г* point, in-plane effective electrons masses along the AC and ZZ directions can be obtained by [42]:

(2) $$m_{AC}=\frac{\hbar^{2}}{2\frac{\gamma^{2}}{\Delta }+\eta_{c}},m_{ZZ}=\frac{\hbar^{2}}{2\upsilon_{c}}$$

For 3-layer BP, the layer-dependent bandgap Δ is 1.1 eV [43]. The other parameters used here are *η_c_* = *ħ*^2^/0.4*m_0_*, *υ_c_* = *ħ*^2^/0.4*m_0_*, and *γ* = 4*a*/*π* eVm. *a* (= 0.223 nm) is the scale length of the BP and *π*/*a* is the width of the Brillouin zone. *m_0_* = 9.10938 × 10^−31^ kg is the standard electron rest mass. The in-plane anisotropic conductivity of BP can be described by a simple semiclassical Drude model. The conductivity of in-plane BP along the AC and ZZ crystalline directions are given as [44]

(3) $$\sigma_{AC,ZZ}=\frac{iD_{AC,ZZ}}{\pi (\omega +i\eta /\hbar )}$$

where *D_AC, ZZ_* = *πe*^2^*ρ*/*m _AC, ZZ_*, is the Drude weight, which is dependent on the electron charge. *η* (= 10 meV) is used to define the BP relaxation rate. *ρ* (= 5 × 10^13^ cm^−2^) is the electron doping. With the angle *φ*, the conductance matrix connecting the surface current and electric field can be expressed as *σ* = [*σ_pp_*, *σ_ps_*; *σ_sp_*, *σ_ss_*], where *σ_pp_* = *σ_AC_*cos2φ + *σ_ZZ_*sin2*φ*, *σ_ss_* = *σ_AC_*sin2*φ* + *σ_ZZ_*cos2*φ*, and *σ_sp_* = *σ_ps_* = (*σ_ZZ_* − *σ_AC_*) sin*φ*cos*φ* [45]. The cross conductivity *σ_sp_* vanishes for isotropic 2D materials such as graphene. Hence, the effective permittivity of BP in *p*-axis and *z*-axis directions can be derived by

(4) $$\left\{ \begin{array}{l} \varepsilon_{pp}=\varepsilon_{r}+\frac{i\sigma_{pp}}{\varepsilon_{0}\omega t_{BP}}\\ \varepsilon_{zz}=\varepsilon_{r} \end{array}\right.$$

where *ε_r_* ( = 5.76) is the relative permittivity of BP [39] and *ε_0_* is the vacuum permittivity. 

Here, the working wavelength is *λ* = 5 μm. Obviously, the length *d* of periodic unit of the BP-based HMMs structure is sufficiently small compared to the working wavelength, so the proposed multilayer structure can be modeled as an anisotropic effective medium by the effective medium theory (EMT) [46]. In *p*–*z* plane, we define the *p*-axis component of effective permittivity as *ε_p_* and the *z*-axis component of effective permittivity as *ε_z_*. Based on the EMT, *ε_p_* and *ε_z_* can be expressed as follows,

(5) $$\left\{ \begin{array}{l} \varepsilon_{p}=\left(t_{BP}\varepsilon_{pp}+t_{d}\varepsilon_{d}\right)/(t_{BP}+t_{d})\\ \varepsilon_{z}=(t_{BP}+t_{d})\varepsilon_{zz}\varepsilon_{d}/(t_{BP}\varepsilon_{d}+t_{d}\varepsilon_{zz}) \end{array}\right.$$

In *k* space, the IFC of BP-based HMMs for *p*-polarized light is given by

(6) $$\frac{k_{p}^{2}}{\varepsilon_{z}}+\frac{k_{z}^{2}}{\varepsilon_{p}}=k_{0}^{2}$$

where *k_p_* and *k_z_* are the components of the wavevector along *p*- and *z*-directions respectively, *k_0_* (= *ω*/*c*) is the wavevector of light in the vacuum. 

**Figure 2:**
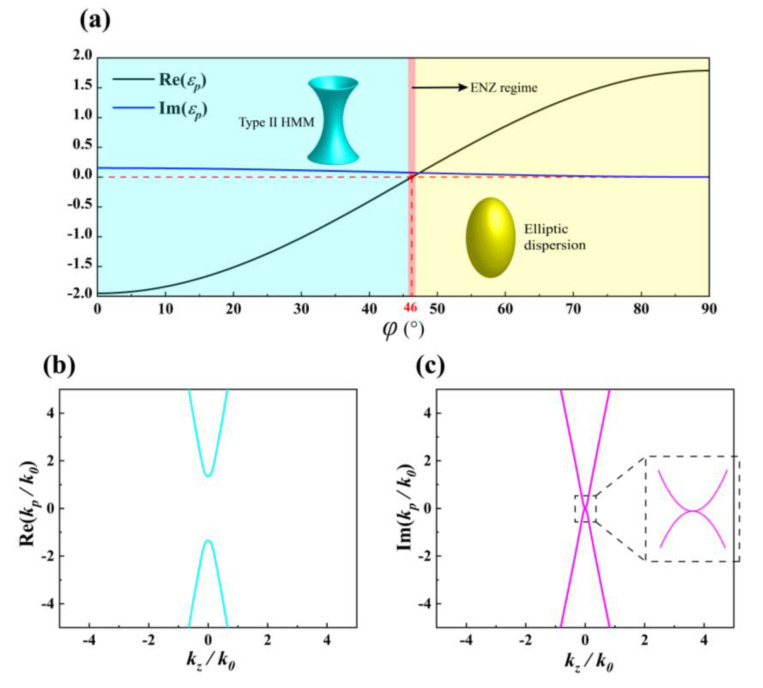
(**a**) Re(*ε_p_*)-*φ* (black line) and Im(*ε_p_*)-*φ* (blue line) curves. Calculated IFC for (**b**) Re(*k_p_*/*k_0_*) and (**c**) Im(*k_p_*/*k_0_*) as a function of *k_z_*/*k_0_*. The other parameters are *λ* = 5 μm, *ρ* = 5 × 10^13^ cm^−2^, and *t_d_* = 300 nm.

According to Equation (5), we calculate the *p*-axis and *z*-axis components of effective permittivity under different *φ* when the working wavelength *λ* is 5 μm. We find that *ε_z_* (= 1.885) is a constant positive value and *ε_p_* is a variable with *φ*. As shown in Figure 2a, Re (*ε_p_*) changes from negative to positive values as *φ* increases from 0° to 90°, and Im (*ε_p_*) is always near 0. In addition, when Re (*ε_p_*) = 0, we can regard it as PTT point. Here, the PTT refers to the optical topological transition of HMMs’ IFC instead of the topological phase transition. When the IFC of structure transitions between open hyperboloid and closed ellipsoid, PTT point will exist. The regime, which is very close to PTT point, is also known as the epsilon-near-zero (ENZ) regime [21]. At PTT point, HMMs can significantly suppress the diffraction and scattering of incident light, which can provide a new way for efficiently manipulating light-matter interactions at nanoscales. For our proposed BP-based HMMs structure, it will change from Type II HMM to elliptic dispersion near PTT point. Based on Equation (6), we calculate the complex wavevector *k_p_*/*k_0_* of BP-based HMMs’ IFC at *φ* = 46°, as shown in Figure 2b,c. For an ideal lossless medium, HMMs’ IFC will degenerate to two points at PTT point [23], which means that only the light with pure wavevectors along *p*-axis is through metamaterials and an angular transparency window is achieved along the *p*-axis direction. Here, for our BP-based HMMs, the topology of IFC at PTT point maintains a narrow hyperboloid as shown Figure 2b. Thus, the light with very small *k_z_* wavevectors are allowed to propagate inside the metamaterials. As shown in Figure 2c, Im (*k_p_*/*k_0_*) as function of *k_z_*/*k_0_* exhibits a conical dispersion. The inset of Figure 2c shows that the conical dispersion achieves a degenerate state at the origin and the light with wavevector along *p*-axis has Im(*k_p_*/*k_0_*) = 0. This indicates that the light with wavevector along *p*-axis will not be affected by absorption losses. However, for the light with small *k_z_* wavevectors, the intrinsic loss of materials will continue to exist due to Im(*k_p_*/*k_0_*) is not close to zero in this moment. In general, even though the light with small *k_z_* wavevectors can propagate inside the metamaterial, the existence of intrinsic loss makes it possible to suppress the light propagation away from *p*-axis direction, which is helpful for maintaining a narrow angular optical transparency window. Based on the above theoretical analysis, our proposed HMMs provides a possible way to realize a narrow angular optical transparency window when the incident *p-*polarized light in *p*-z plane due to PTT. 

## 3. Results and Discussion

**Figure 3:**
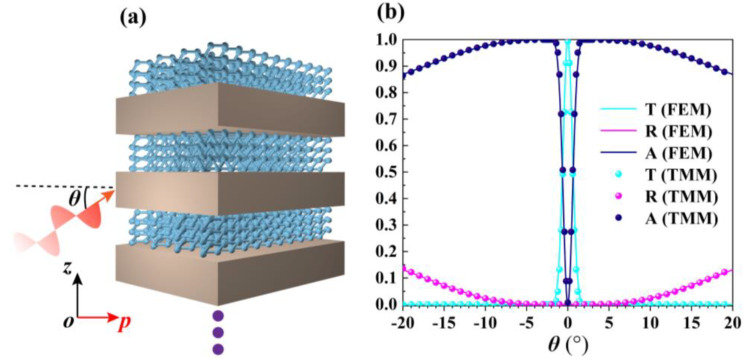
(**a**) The *p*-*z* view of the proposed BP-based HMMs structure and a *p*-polarized light is incident on the structure at an incident angle *θ*. (**b**) Transmission (*T*), reflection (*R*), and absorption (*A*) of BP-based HMMs structure under *λ* = 5 μm, *ρ* = 5 × 10^13^ cm^−2^, and *t_d_* = 300 nm. The curve (dots) are numerical (theoretical) results obtained by the FEM (TMM).

As shown in Figure 3a, a *p*-polarized light is incident on the BP-based HMMs structure at an incident angle *θ* in the *p*-*z* plane. Here, the angle between the *p*-*z* and *y-z* plane is *φ* = 46° and working wavelength *λ* = 5 μm. We simulate the transmission, reflection, and absorption of the multilayer structure with 138 μm width along the *p*-axis. In this work, all numerical simulation results are obtained by the commercial software COMSOL Multiphysics (COMSOL Multiphysics 5.4, Stockholm, Sweden) based on FEM. In our work, periodic boundary conditions are adopted in the *z*-axis direction. We verify the position of PTT point by EMT. In addition, we theoretically analyze the propagation features of the proposed BP-based HMMs structure by using transfer matrix method (TMM) under *p*-polarized light. Based on Maxwell’s equations and boundary conditions, the magnetic field between adjacent layers can be related via a transfer matrix, which can be obtained as follows [47]

(7) $$M_{i}(\theta ,d_{i})=\left[\begin{array}{cc} \cos(k_{ip}d_{i}) & -jq_{ip}\sin(k_{ip}d_{i})\\ -j\frac{1}{q_{ip}}\sin(k_{ip}d_{i}) & \cos(k_{ip}d_{i}) \end{array}\right]$$

Here, we divide BP-based HMMs structure into *k* layers along the *p*-axis direction. The subscript *i* corresponds to the propagation of light in the *i*-th layer with a thickness of *d_i_* and *q_p_* = *k_p_*/(*k_0_*)*ε_z_*. According to Equation (6), we can get $k_{p}=\sqrt{\varepsilon_{z}k_{0}^{2}-(\varepsilon_{z}/\varepsilon_{p})k_{z}^{2}}$. Here, *k_z_* (= *k_0_*sin*θ*) is the *z* component of incident light wavevector. The total transfer matrix (*M*(*θ*)) connecting the fields at the incident end and the exit end can be expressed as

(8) $$\left[\begin{array}{cc} M_{11}(\theta ) & M_{12}(\theta )\\ M_{21}(\theta ) & M_{22}(\theta ) \end{array}\right]=\prod_{i=1}^{n}M_{i}(\theta ,d_{i})$$

By means of the TMM, the reflection and transmission coefficients can be calculated as

(9) $$r(\theta )=\frac{q_{0}(M_{11}(\theta )-M_{22}(\theta ))+q_{0}^{2}M_{12}(\theta )-M_{21}(\theta )}{q_{0}(M_{11}(\theta )+M_{22}(\theta ))+q_{0}^{2}M_{12}(\theta )+M_{21}(\theta )}$$

(10) $$t(\theta )=\frac{2}{M_{11}(\theta )+M_{22}(\theta )+q_{0}M_{12}(\theta )+(1/q_{0})M_{21}(\theta )}$$

where *q_0_* = cos*θ*, the reflection (*R*(*θ*)) and transmission (*T*(*θ*)) of the structure can be obtained by |*r*(*θ*)|^2^ and |*t*(*θ*)|^2^, respectively. Further, the absorption can be written as *A*(*θ*) = 1−*R*(*θ*)−*T*(*θ*).

Figure 3b depicts the FEM-simulated and TMM-calculated optical transmission (*T*), reflection (*R*) and absorption (*A*) of *p*-polarized light as a function of incident angle (*θ*) for the proposed BP-based HMMs structure when *φ* = 46°. Obviously, the results obtained by numerical simulation (FEM) are in good agreement with the theoretical calculation (TMM). The results indicate that a narrow angular optical transparency window with angular full width at half maximum (FWHM) of 1.32° appears. The transmittance can reach 99.7% at *θ* = 0°, but the transmittance is only 0.1% at *θ* = 2°. The reason is that the incident light will be affected by the material’s inherent losses and the energy will be attenuated with further propagation in the medium except for the wavevector of light along *p*-axis direction. Moreover, the absorption in the Figure 3b drops to almost zero when *θ* = 0°, which can also verify the results obtained in Figure 2c. So, the PTT point of proposed BP-based HMMs structure happens when *φ* = 46°, working wavelength *λ* = 5 μm, electron doping *ρ* = 5 × 10^13^ cm^−2^, and the thickness of SiO_2_ *t_d_* = 300 nm.

**Figure 4:**
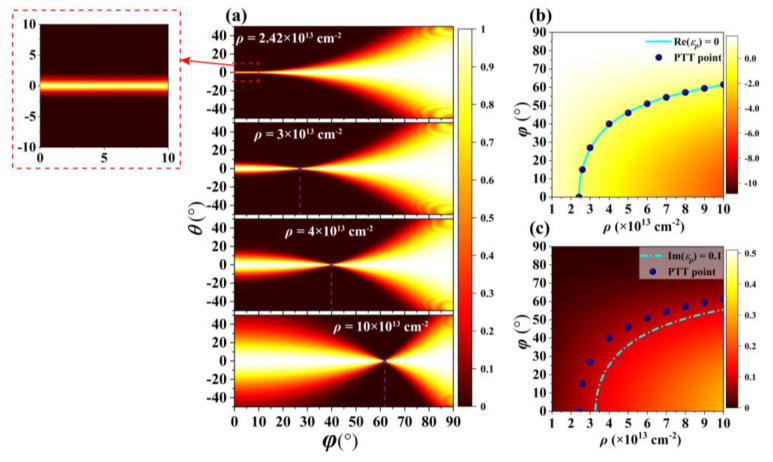
(**a**) Numerically simulated optical transmission as a function of the angle *φ* and incident angle *θ* when *ρ* = 2.42 × 10^13^, 3 × 10^13^, 4 × 10^13^, and 10 × 10^13^ cm^−2^, respectively. (**b**) Re(*ε_p_*) and (**c**) Im(*ε_p_*) as function of *ρ* and *φ*.

Then, the influence of electron doping of BP on the position of PTT point for the proposed BP-based HMMs structure will be discussed. Figure 4a shows numerically simulated optical transmission as a function of the angle *φ* between the incidence plane (*p*-*z* plane) and *y*-*z* plane, and incident angle *θ* when the electron doping of BP is 2.42 × 10^13^, 3 × 10^13^, 4 × 10^13^, and 10 × 10^13^ cm^−2^, respectively. PPT happens when the narrowest angular optical transparency window is achieved. Obviously, the angle *φ*, which is corresponding to the PTT, is increasing from 0° to 61° as the electron doping of BP increases from 2.42 × 10^13^ to 10 × 10^13^ cm^−2^ when the other parameters unchanged. The FWHMs for narrowest angular optical transparency window are all smaller than 1.328° in Figure 4a. Figure 4b,c show Re(*ε_p_*) and Im(*ε_p_*) as a function of electron doping *ρ* of BP and angle *φ* based on Equation (5), respectively. Here, the purple dots represent the position of the PTT point obtained by simulation calculation. The cyan solid line in the Figure 4b represents Re (*ε_p_*) = 0, and the cyan dashed line in the Figure 4c represents Im (*ε_p_*) = 0.1. We find that these purple dots which are obtained by simulation satisfy Re (*ε_p_*) ≈ 0 and Im (*ε_p_*) < 0.1. It means that the PTT point obtained by simulation calculation is consistent with that obtained by theoretical calculation. Besides, as shown in the inset of Figure 4a, we can also achieve a wide range of narrow angular optical transparency window from *φ* = 0° to *φ* = 10° due to the existence of ENZ regime when *ρ* = 2.42 × 10^13^ cm^−2^, and the FWHM of the angular optical transparency window stays around 1.3°.

**Figure 5:**
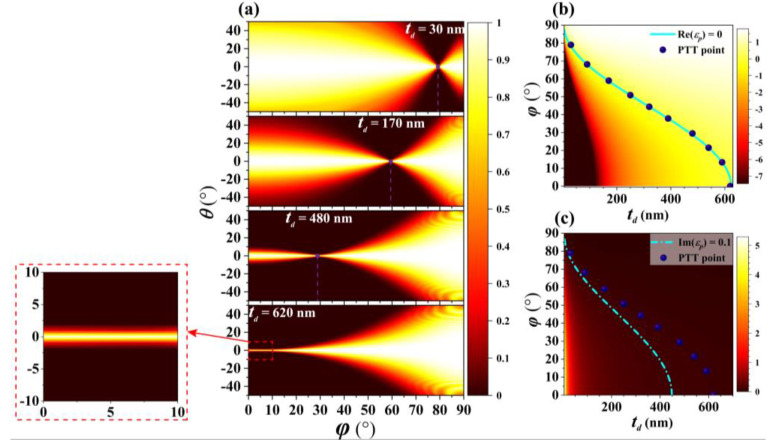
(**a**) Numerically simulated optical transmission as a function of the angle *φ* between the incidence plane (*p*-*z* plane) and *y*-*z* plane, and incident angle *θ* when *t_d_* = 30, 170, 480, and 620 nm, respectively. (**b**) Re(*ε_p_*) and (**c**) Im(*ε_p_*) as function of *t_d_* and *φ*.

The influence of the thickness *t_d_* of SiO_2_ on the position of PTT point is also discussed in detail. Figure 5a shows numerically simulated optical transmission as a function of the angle *φ* between the incidence plane (*p*-*z* plane) and *y*-*z* plane, and incident angle *θ* when the thickness of SiO_2_ is 30, 170, 480, and 620 nm, respectively. The FWHMs for narrowest angular optical transparency window are all smaller than 1.334° in Figure 5a. The angle *φ*, which is corresponding to the PTT point, is decreasing from 79° to 0° as the thickness of SiO_2_ increases from 30 to 620 nm when the other parameters unchanged. Figure 5b,c show Re(*ε_p_*) and Im(*ε_p_*) as a function of *t_d_* and angle *φ* based on Equation (5), respectively. Here, the purple dots represent the position of the PTT point obtained by simulation calculation. The cyan solid line in the Figure 5b represents Re (*ε_p_*) = 0, and the cyan dashed line in the Figure 5c represents Im (*ε_p_*) = 0.1. It is found that these purple dots obtained by simulation satisfy Re (*ε_p_*) ≈ 0 and Im (*ε_p_*) < 0.1. It means that the PTT point obtained by simulation calculation agrees with that obtained by theoretical calculation. Besides, as shown in the inset of Figure 5a, a wide range of narrow angular optical transparency window is also achieved from *φ* = 0° to *φ* = 10° due to the existence of ENZ regime when *t_d_* = 620 nm.

**Figure 6:**
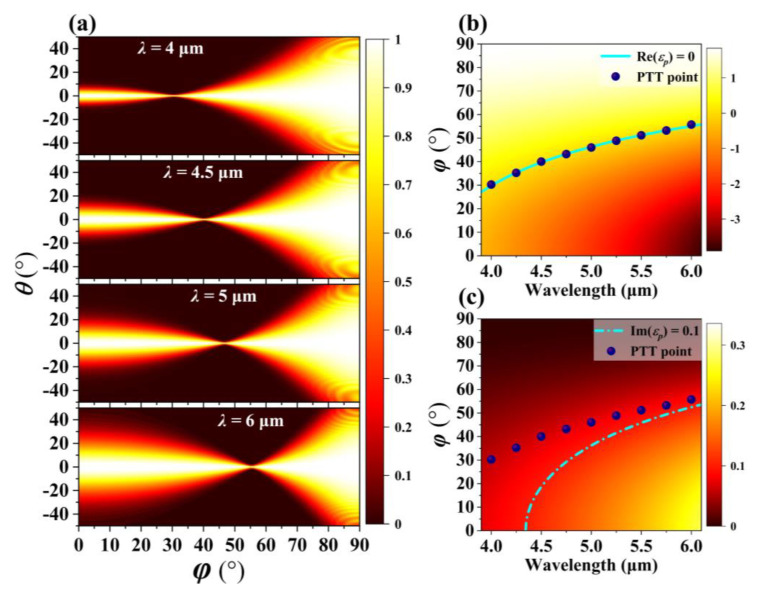
(**a**) Numerically simulated optical transmission as a function of the angle *φ* between the incidence plane (*p*-*z* plane) and *y*-*z* plane, and incident angle *θ* when *λ* = 4, 4.5, 5, and 6 μm, respectively. (**b**) Re(*ε_p_*) and (**c**) Im(*ε_p_*) as function of *λ* and *φ*.

Finally, we discuss the influence of working wavelength *λ* on the position of PTT point for the proposed BP-based HMMs structure. Figure 6a shows numerically simulated optical transmission as a function of the angle *φ* between the incidence plane (*p*-*z* plane) and *y*-*z* plane, and incident angle *θ* when working wavelength *λ* is 4, 4.5, 5, and 6 μm, respectively. The FWHM for narrowest angular optical transparency window is approximately 1.3° in Figure 6a. Obviously, the angle *φ*, which is corresponding to the PTT point, is increasing from 30° to 56° as *λ* increases from 4 to 6 μm when the other parameters unchanged. Figure 6b,c show Re(*ε_p_*) and Im(*ε_p_*) as a function of *λ* and angle *φ* based on Equation (5), respectively. Here, the purple dots represent the position of the PTT point obtained by simulation calculation. The cyan solid line in the Figure 6b represents Re (*ε_p_*) = 0, and the cyan dashed line in the Figure 6c represents Im (*ε_p_*) = 0.1. These purple dots obtained by simulation satisfy Re (*ε_p_*) ≈ 0 and Im (*ε_p_*) < 0.1. Thus, the PTT point obtained by simulation calculation is consistent with that obtained by theoretical calculation. 

## 4. Conclusions

In summary, anisotropic PTT is theoretically and numerically investigated based on the proposed BP-based HMMs structure consisting of alternating BP/SiO_2_ multilayer. By tailoring the IFC of BP-based HMMs from open hyperboloid to closed ellipsoid, both the theoretical calculations and numerical simulations show that a narrow angular transparency window appears at PTT point for *p*-polarized light. Moreover, we find that the angle *φ*, at which PTT appears, can be affected by working wavelength *λ*, thickness *t_d_* of SiO_2_, or electron doping *ρ* of BP. It is believed that our work provides a new way in various angle-dependent optical applications, such as privacy protection and detectors with ultra-high signal-to-noise ratios.
